# Proton pump inhibitors and potassium competitive acid blockers decrease pembrolizumab efficacy in patients with metastatic urothelial carcinoma

**DOI:** 10.1038/s41598-024-53158-1

**Published:** 2024-01-30

**Authors:** Keitaro Iida, Taku Naiki, Toshiki Etani, Takashi Nagai, Yosuke Sugiyama, Teruki Isobe, Maria Aoki, Satoshi Nozaki, Yusuke Noda, Nobuhiko Shimizu, Nami Tomiyama, Masakazu Gonda, Hiroyuki Kamiya, Hiroki Kubota, Akihiro Nakane, Ryosuke Ando, Noriyasu Kawai, Takahiro Yasui

**Affiliations:** 1 Department of Nephro-urology, Graduate School of Medical Sciences, Nagoya City University, Kawasumi 1, Mizuho-cho, Mizuho-ku, Nagoya, 467-8601 Japan; 2 Department of Urology, Gamagori City Hospital, Gamagori City, Japan; 3 Department of Pharmacy, Nagoya City University Hospital, Nagoya, Japan; 4 Department of Urology, Anjo Kosei Hospital, Anjo City, Japan; 5 Department of Urology, Toyota Kosei Hospital, Toyota City, Japan; 6 Department of Urology, Daido Hospital, Nagoya City, Japan; 7 Department of Urology, Kainan Hospital, Yatomi City, Japan; 8 Department of Education and Research Center for Community Medicine, Nagoya City University Graduate School of Medical Sciences, Nagoya, Japan

**Keywords:** Cancer, Urological cancer, Bladder cancer, Immunotherapy

## Abstract

We elucidated the efficacy of gut microbiome–altering drugs on pembrolizumab efficacy in patients with metastatic urothelial carcinoma (mUC). Clinical data were analyzed retrospectively from 133 patients with mUC who received second-line pembrolizumab therapy between January 2018 and January 2021, following failed platinum-based chemotherapy. We evaluated the effects of gut microbiome–altering drugs (proton pump inhibitors [PPI]/potassium-competitive acid blockers [P-CAB], H2 blockers, antibiotics, non-steroidal anti-inflammatory drugs [NSAIDs], metformin, antipsychotics, steroids, and opioids), taken by patients within 30 days before/after pembrolizumab treatment, on progression-free survival (PFS) and overall survival (OS). Fifty-one patients received PPI/P-CAB (37/14, respectively); H2 blockers, 7; antibiotics, 35; NSAIDs, 22; antipsychotics, 8; metformin, 3; steroids, 11; and opioids, 29. Kaplan–Meier curves revealed PPI or P-CAB users showed shorter PFS than non-PPI-P-CAB users (*p* = 0.001, *p* = 0.005, respectively). Multivariate analysis highlighted PPI/P-CAB use as the only independent prognostic factor for disease progression (hazards ratio: 1.71, 95% confidence interval: 1.14–2.07, *p* = 0.010) but not death (*p* = 0.177). Proton pump inhibitors/potassium-competitive acid blockers may decrease the efficacy of pembrolizumab therapy for mUC, possibly via gut microbiome modulation.

## Introduction

Immune checkpoint inhibitors (ICI) have been used to treat a variety of cancers since 2010. In 2017, the US Food and Drug Administration approved pembrolizumab and atezolizumab for second-line chemotherapy following platinum based-first-line chemotherapy against urothelial carcinoma. However, very little attention has been paid to the relationship between drug taking and ICI treatment in the literature. Recently, a role has been revealed for the gut microbiome in cancer treatment, especially when using ICI. Gopalakrishnan et al.^1^ investigated features of the gut microbiome in patients with melanoma who underwent anti–programmed cell death protein 1 (PD-1) therapy and found that responders showed a higher alpha diversity. Antibiotics are known to induce dysbiosis of the gut microbiome^2^. This may have a negative effect on the efficacy of PD-1 or programmed death-ligand 1 (PD-L1) monoclonal antibodies (mAb) against several cancers, such as renal cell, non-small-cell lung, melanoma, and urothelial cancers^3–6^. Potassium competitive acid blockers (P-CAB) and proton pump inhibitors (PPI) can also induce dysbiosis of the gut microbiome, consequently increasing the risk of *Clostridium difficile* infection^7,8^. Several reports described how PPI affected the efficacy of PD-1/PD-L1 mAb^9–15^, which seemed to differ according to cancer type, drugs, and patient ethnicities. Most of the reports described the efficacy of ICI with single drug use, but did not comprehensively investigate gut microbiome–altering drugs. Here, we highlight how the aforementioned gut microbiome–altering drugs affect the efficacy of PD-1 mAb (pembrolizumab) as second-line therapy in Japanese patients with metastatic urothelial carcinoma (mUC).

## Methods

### Patient enrollment

We performed a retrospective study of 143 patients with pembrolizumab used as second-line therapy or beyond for advanced or metastatic urothelial carcinoma at Nagoya City University Hospital plus six affiliated hospitals from December 2018 to February 2021. Pembrolizumab was used at either 200 mg every 3 weeks or 400 mg every 6 weeks. Histories of gut microbiome–altering drugs administered either orally or intravenously within 30 days, and prior to the initiation of pembrolizumab, were collected from patients’ records. The following were employed as gut microbiome–altering drugs: PPI/P-CAB, H2 blockers, antibiotics, antipsychotics, non-steroidal anti-inflammatory drugs (NSAIDs), metformin, steroids, and opioids^16^. All the steroid users took a low-dose steroid (less than 40 mg/body daily prednisolone or other steroids equivalent to prednisolone). The items of opioids consisted of weak opioids such as tramadol or tapentadol as well as strong ones. After the first pembrolizumab administration, patients were evaluated within 2 months by computer tomography (CT) scan except for patients who died within 2 months. After the initial evaluation, follow-up CT scans were done every 2–3 months. Adverse events (AEs) were determined according to Common Terminology Criteria for Adverse Events (CTCAE) version 5.0. Neutrophil, lymphocyte, hemoglobin, and albumin data were obtained within 2 weeks prior to the start of pembrolizumab therapy.

### Statistical analysis

Progression-free survival (PFS) was defined as either disease progression on a CT scan determined by Response Evaluation Criteria in Solid Tumors version 1.1 or death. Overall survival (OS) was measured from the date of administration of pembrolizumab to death. Differences found in categorical parameters were compared using a *t*-test, and Mann–Whitney *U*, Kruskal Wallis, and Fisher’s exact tests. A Kaplan–Meier method was used for survival curves that were compared using a log-rank test for univariate analysis. Multivariate analysis using Cox’s proportional hazards model was used to examine the effects of pembrolizumab. The following variables were analyzed: age, primary site, sex, treatment lines, Eastern Cooperative Oncology Group (ECOG) performance status (PS), liver metastasis, neutrophil-to-lymphocyte ratio (NLR), albumin, hemoglobin, and gut microbiome–altering drugs as above. Cut-off values for the NLR were as previously described^15,17^. When analyzing the survival of three groups, we evaluated *p*-values by analysis of variance in consideration of multiplicity. If the null hypothesis was rejected, multiplicity’s adjustment of *p*-values was not performed at pairwise log-rank test (post hoc Bonferroni test). EZR software was used for statistical analyses (Saitama Medical Center, Jichi Medical University, Yakushiji, Japan). Our institutional research ethics committee approved this study (Nagoya City University ethical board No. 60-18-0060). All methods were performed according to the relevant guidelines, and informed consent was obtained from all study participants. The design of the investigation was according to the Declaration of Helsinki (2013).

## Results

### Patients’ characteristics and oncological outcomes

Of 143 patients with mUC for whom pembrolizumab was used as subsequent second-line therapy, a total of 133 consecutive patients met eligibility criteria. Ten patients were excluded: two due to the cessation of pembrolizumab for non-clinical reasons, four due to missing data, and four due to non-urothelial carcinoma (two adenocarcinomas, one squamous carcinoma, and one unknown). In the total cohort, the median follow-up period was 6.6 months (range 0.5–36.4 months). Of the gut microbiome–altering drugs described above, the most abundant drugs included PPI/P-CAB prescribed for 51 patients (37/14, respectively), followed by H2 blockers for 7, antibiotics for 35 (systemic antibiotic treatment for more than 8 days for 14), NSAIDs for 20, metformin for 4, antipsychotics for 8, steroids for 11, and opioids for 29. Patients were classified as a PPI/P-CAB user or not and their data then statistically analyzed. The characteristics of PPI/P-CAB users and non-users are listed in Table 1. Of the patients’ basic clinical variables, including median age, ECOG-PS, and distribution of metastatic sites were not statistically different between two groups. Reflecting the clinical course, the PPI/P-CAB group had a significantly higher number of users of the gut-microbiome–altering steroids and opioids and a lower hemoglobin level compared to the non-PPI/P-CAB group. The best responses of patients in the PPI/P-CAB group were: complete response (CR), 2; partial response (PR), 5; stable disease (SD), 6; and progressive disease (PD), 38. For the non-PPI/P-CAB group, responses were: CR, 7; PR, 23; SD, 16; and PD, 36. Progression was evident in 45 (88.2%) patients of the PPI/P-CAB group and in 57 (69.5%) patients of the non-PPI/P-CAB group, respectively. Death occurred in 21 (41.2%) patients of the in PPI/P-CAB group and in 45 (54.9%) patients of the non-PPI/P-CAB group, respectively. Thus, the PPI/P-CAB group showed more disease progression and death than the non-PPI/P-CAB group.

**Table 1:** Patients’ backgrounds showing PPI/P-CAB and non-PPI/P-CAB users.

Characteristics		Non-PPI/P-CAB user group (n = 82)	PPI/P-CAB user group (n = 51)	*p* value
Median age, years (range)		72 (39–85)	73 (48–87)	0.447^†^
Gender, n (%)	Male	69 (84)	34 (67)	0.032^††^
	Female	13 (16)	17 (33)	
Primary site, n (%)	Bladder	40 (49)	22 (43)	0.309^††^
	Upper urinary tract	32 (39)	26 (51)	
	Both	10 (12)	3 (6)	
Treatment lines of ICI, n (%)	2nd line	61 (74)	33 (65)	0.246^††^
	3rd line later	21 (26)	18 (35)	
ECOG–PS, n (%)	0, 1	66 (80)	37 (73)	0.295^††^
	≥ 2	16 (20)	14 (27)	
Metastatic site, n (%)	Lymph node only	24 (29)	12 (24)	0.702^††^
	Existence of liver metastasis	16 (20)	14 (27)	
	other	42 (51)	25 (49)	
H2 blocker, n (%)		6 (7)	1 (2)	0.250^††^
Antibiotics, n (%)		19 (22)	16 (31)	0.317^††^
NSAIDs, n (%)		14 (16)	6 (12)	0.338^††^
Metformin, n (%)		4 (5)	0 (0)	0.298^††^
Antipsychotic, n (%)		4 (5)	4 (8)	0.482^††^
Steroid, n (%)		3 (3)	8 (16)	0.022^††^
Opioid, n (%)		11 (13)	18 (35)	0.005^††^
Median NLR levels, (range)		3.2 (0.8–26.8)	3.6 (0.7–27.6)	0.078^†††^
Median albumin level, g/dL (range)		3.7 (2.2–4.7)	3.5 (2.1–4.4)	0.167^†††^
Median Hb levels, g/dL (range)		10.8 (7.0–15.8)	10.0 (7.4–15.9)	0.046^†††^

ECOG-PS, eastern cooperative oncology group performance status; Hb, hemoglobin; ICI, immune checkpoint inhibitors; NLR, neutrophil-to-lymphocyte ratio; NSAIDs, non-steroidal anti-inflammatory drugs; PPI/P-CAB, proton pump inhibitors/potassium-competitive acid blockers.

^†^t-test, ^††^Fisher's exact test, ^†††^Mann–Whitney U test.

### Univariate and multivariate analyses for predicting risk factors concerning PFS and OS

**Figure 1:**
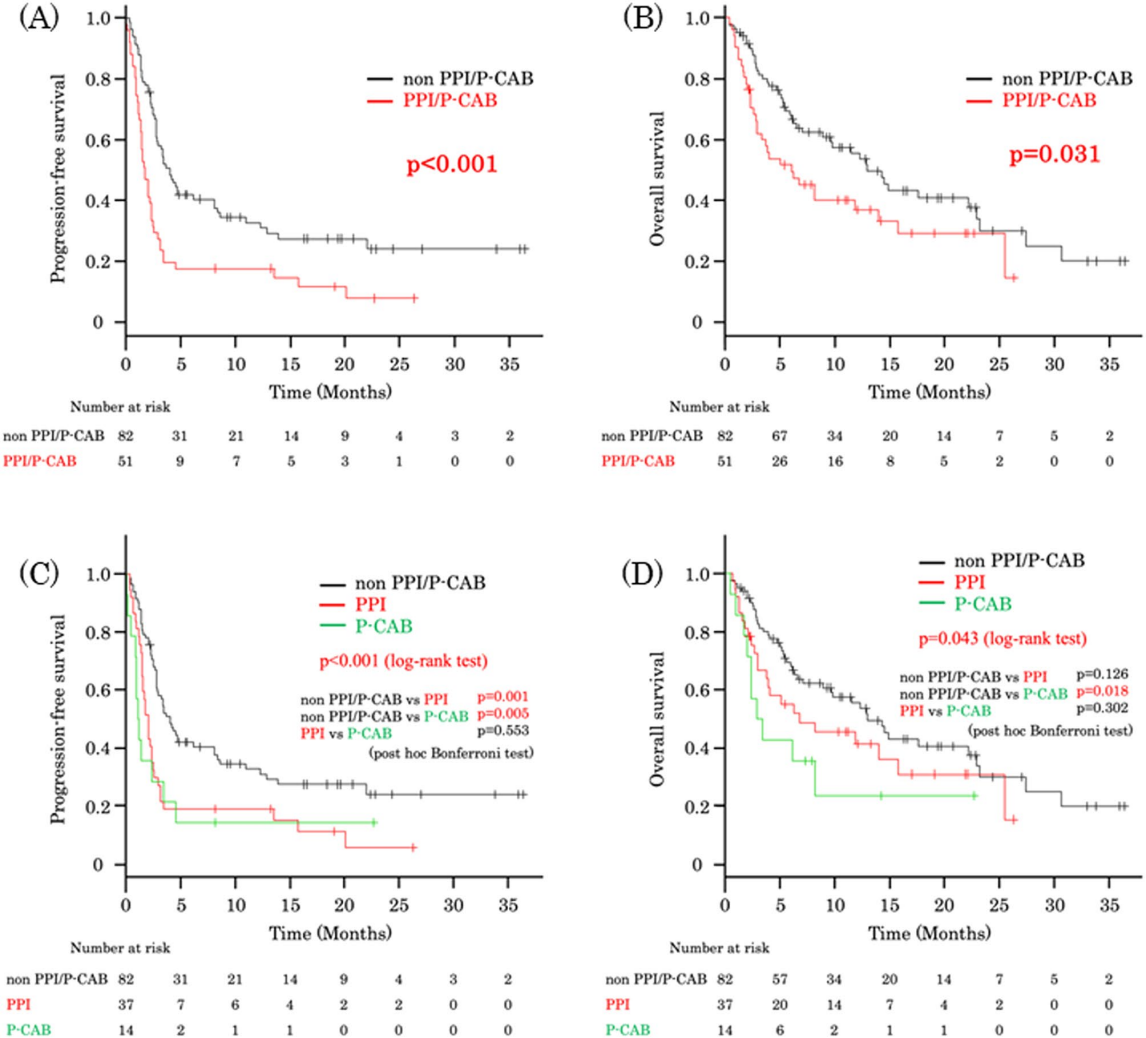
Kaplan–Meier curves for patients treated with pembrolizumab, with and without the use of PPI or P-CAB. PPI/P-CAB users showed significantly shorter progression-free survival (**A**) (*p* < 0.001) and overall survival (**B**) (*p* = 0.031). Kaplan–Meier curves for patients treated with pembrolizumab, with or without the use of PPI or P-CAB. PPI and P-CAB users showed significantly shorter progression-free survival than non-users (**C**) (*p* = 0.001, *p* = 0.005, respectively). P-CAB users showed shorter overall survival than non-PPI/P-CAB users (*p* = 0.018). However, no difference in overall survival was noted between PPI and non-PPI/P-CAB users (**D**) (*p* = 0.126). P-CAB, potassium-competitive acid blockers; PPI, proton pump inhibitors.

The median PFS was 1.8 months (95% confidence interval [CI] 1.4–2.4) in the PPI/P-CAB group and 4.1 months (95% CI 2.8–8.1) in the non-PPI/P-CAB group. The median OS was 6.1 months (95% CI 3–14) in the PPI/P-CAB group and 13 months (95% CI 8.9–22.9) in the non-PPI/P-CAB group. Kaplan–Meier curves showed a significant difference between PPI/P-CAB and non-PPI/P-CAB groups with regard to PFS (*p* < 0.001) and OS (*p* = 0.031; Fig. 1A,B), with the former showing shorter PFS and OS.

Univariate analysis showed that ECOG–PS, liver metastasis, PPI/P-CAB, steroids, opioids, NLR, serum albumin levels, and hemoglobin were significantly associated with disease progression. Multivariate analysis revealed that PPI/P-CAB, ECOG–PS, liver metastasis, and NLR were significant and independent prognostic factors for associated disease progression (Table 2). In addition, multivariate analyses of variables to predict OS showed that ECOG–PS, liver metastasis, opioids and the serum albumin level were also independent risk factors, while a history of PPI/P-CAB intake was not (Table 3).

**Table 2:** Uni- and multivariate analyses predicting progression-free survival for patients with metastatic urothelial carcinoma treated with pembrolizumab as second-line treatment.

Parameters	Univariate			Multivariate		
	HR	95% CI	*p* value	HR	95% CI	*p* value
Age at initiation of treatment	1.00	0.98–1.02	0.916	–	–	–
Gender, female vs. male	1.23	0.77–1.97	0.380	–	–	–
Primary site, UTUC vs. bladder	1.11	0.83–1.50	0.463	–	–	–
ECOG-PS, 2 vs. 0, 1	2.91	1.86–4.53	< 0.001	2.31	1.38–3.85	0.001
Treatment lines of ICI, 3rd line later vs. 2nd line	1.16	0.76–1.77	0.488	–	–	–
Liver metastasis, yes vs. no	2.39	1.53–3.73	< 0.001	1.92	1.16–3.17	0.011
PPI/P-CAB	2.06	1.39–3.06	< 0.001	1.71	1.14–2.07	0.010
H2 blockers	1.39	0.61–3.17	0.439	–	–	–
Antibiotics	1.35	0.88–2.10	0.173	–	–	–
NSAIDs	0.99	0.60–1.66	0.982	–	–	–
Metformin	1.66	0.52–5.26	0.389	–	–	–
Antipsychotics	1.42	0.62–3.26	0.403	–	–	–
Steroids	3.47	1.82–6.62	< 0.001	1.63	0.79–3.37	0.188
Opioids	2.39	1.54–3.72	< 0.001	1.30	0.82–2.07	0.266
NLR, ≥ 3.0 vs. < 3.0	1.97	1.31–2.96	0.001	1.44	0.92–2.27	0.111
Serum Alb levels	0.52	0.37–0.72	< 0.001	0.68	0.44–1.04	0.078
Hb levels	0.87	0.78–0.98	0.018	0.89	0.77–1.03	0.122

Alb, albumin; CI, confidence interval; ECOG-PS, Eastern Cooperative Oncology Group Performance Status; Hb, hemoglobin; HR, hazard ratio; ICI, immune checkpoint inhibitors; NLR, neutrophil-to-lymphocyte ratio; NSAIDs, non-steroidal anti-inflammatory drugs; PPI/P-CAB, proton pump inhibitors/potassium-competitive acid blockers; UTUC, upper urinary tract urothelial carcinoma.

**Table 3:** Uni- and multivariate analyses predicting overall survival for patients with metastatic urothelial carcinoma with pembrolizumab as second-line treatment.

Parameters	Univariate			Multivariate		
	HR	95% CI	*p* value	HR	95% CI	*p* value
Age at initiation of treatment	1.01	0.99–1.03	0.433	–	–	–
Gender, female versus male	0.87	0.51–1.51	0.625	–	–	–
Primary site, UTUC versus bladder	0.88	0.62–1.26	0.501	–	–	–
ECOG-PS, 2 versus 0, 1	5.19	3.20–8.40	< 0.001	3.87	2.20–6.79	< 0.001
Treatment lines of ICI, 3rd line later versus 2nd line	1.11	0.69–1.79	0.674	–	–	–
Liver metastasis, yes versus no	3.34	2.05–5.43	< 0.001	2.56	1.48–4.44	< 0.001
PPI/P-CAB	1.64	1.04–2.59	0.033	1.39	0.86–2.24	0.177
H2 blockers	2.06	0.89–4.77	0.093	–	–	–
Antibiotics	1.43	0.86–2.36	0.168	–	–	–
NSAIDs	1.25	0.71–2.20	0.444	–	–	–
Metformin	1.24	0.39–3.98	0.715	–	–	–
Antipsychotics	1.16	0.36–3.70	0.805	–	–	–
Steroids	3.85	1.88–7.88	< 0.001	1.18	0.52–2.67	0.685
Opioids	3.79	2.32–6.19	< 0.001	1.94	1.14–3.28	0.014
NLR, ≥ 3.0 versus < 3.0	2.10	1.31–3.37	0.002	1.16	0.68–1.97	0.589
Serum Alb levels	0.37	0.25–0.54	< 0.001	0.40	0.24–0.67	< 0.001
Hb levels	0.85	0.75–0.97	0.019	0.91	0.76–1.09	0.311

Alb, albumin; CI, confidence interval; ECOG-PS, Eastern Cooperative Oncology Group Performance Status; Hb, hemoglobin; HR, hazard ratio; ICI, immune checkpoint inhibitors; NLR, neutrophil-to-lymphocyte ratio; NSAIDs, non-steroidal anti-inflammatory drugs; PPI/P-CAB, proton pump inhibitors/potassium-competitive acid blockers; UTUC, upper urinary tract urothelial carcinoma.

As shown, PPI/P-CAB was a strong risk factor for disease progression. We subsequently analyzed the effect of PPI or P-CAB on the efficacy of pembrolizumab. The characteristics of PPI or P-CAB users and non-users are listed in Supplementary Table 1. Kaplan–Meier curves of PFS and OS between the three groups (non-PPI/PCAB, PPI, and P-CAB) are also shown (Fig. 1C,D). Significant differences were found in PFS between non-PPI/PCAB and PPI groups, and non-PPI/PCAB and P-CAB groups (*p* = 0.001, *p* = 0.005, respectively, post hoc Bonferroni test). There was also a significant difference in OS between non-PPI/PCAB and P-CAB groups (*p* = 0.018, post hoc Bonferroni test), while no difference was observed between non-PPI/PCAB and PPI groups (*p* = 0.126, post hoc Bonferroni test). Thus, the non-PPI/P-CAB group showed significantly longer PFS and OS compared to the PPI group.

Multivariate analysis revealed that the PPI group was significantly associated with disease progression but the P-CAB group was not (Supplementary Table 2; *p* = 0.018). However, multivariate analyses of variables to predict OS showed that neither PPI nor P-CAB groups were independent risk factors (Supplementary Table 3).

We further explored the effect of time period over which antibiotics were used; a short period of use was designated as less than or equal to 7 days, such as for the treatment of cystitis, and a long period of use was defined as more than or equal to 8 days, such as for the treatment of pneumonia or pyelonephritis. Univariate analysis revealed that for a long period of antibiotic use, significantly longer PFS and OS were noted compared to non-antibiotic users (HR 2.13, 95% CI 1.16–3.91, *p* = 0.014, HR 2.04, 95% CI 1.01–4.10, *p* = 0.047). However, multivariate analysis did not show a long period of antibiotic use as being a significant risk factor in predicting disease progression or death (Supplementary Table 3).

### Adverse effects

Subsequently, we analyzed the relationship between gut microbiome–altering drugs and gastrointestinal adverse events during pembrolizumab administration. None of the aforementioned drugs were associated with any stage of diarrhea, while constipation was experienced by PPI/P-CAB users only, as opposed to non-PPI/P-CAB users (*p* = 0.02). No patients experienced Grade 3 or 4 constipation; all three patients who developed Grade 3 or 4 diarrhea were non–PPI/P-CAB users.

## Discussion

Although the prognosis for patients with various types of cancers has improved noticeably since the launch of ICI, not all patients have benefitted from their use. With respect to patients with mUC treated with pembrolizumab, a quarter showed a CR or PR but the rest showed no response to treatment^18^.

The use of PPI is known to induce dysbiosis and predispose patients to Clostridium difficile infection^19,20^. Additionally, PPI were noted to reduce the diversity of the gut microbiome and increase the number of species of resident oral microbiota in non-cancer patients^21^. Moreover, PPI induced an increase in the genera, Bacteroides, Streptococcus, and Rothia^8,21^. In addition to these genera, P-CAB, which was 400 times more potent in inhibiting gastric acid secretion than PPI^22^, was also described as inducing an increase in the genus, Actinomyces^8^.

As shown in Table 4, several reports exist that describe a relationship between a history of taking PPI and the effect of ICI^9–15^. Hopkins et al.^14^ and Ruiz-Banobre et al.^15^ showed how the use of PPI reduced PFS and OS in patients with mUC treated with ICI. Of these studies, the influence of either PPI, or PPI and antibiotics, on the efficacy of pembrolizumab was examined using such gut microbiome–altering drugs. Reports demonstrating the effect of gut microbiome–altering drugs on the efficacy of ICI against several malignancies revealed controversial results^9–11^. In comparison, studies on UC and non-small cell lung cancer indicated a worse prognostic outcome for PPI users compared to non-PPI users^12–15^. Spakowicz et al.^10^ and Buti et al.^11^ demonstrated how steroids, in addition to PPI and antibiotics, affected the efficacy of ICI on cancers. The rest of the studies listed in Table 4 did not take into consideration other gut microbiome–altering drugs, besides PPI or antibiotics^9,12–15^. Moreover, the effect of the use of P-CAB on the efficacy of ICI has never previously been reported. In our study, the Kaplan–Meier curves of the PPI and P-CAB groups were almost similar; they also both showed shorter PFS than the non-PPI/P-CAB group. However, multivariate analysis revealed that PPI was an independent factor that predicted disease progression but P-CAB was not. This disparity might be because our study had a smaller number of patients who took P-CAB compared to PPI. Nevertheless, our study provides a new insight into the negative effect of the use of PPI/P-CAB, among other gut microbiome–altering drugs, on ICI treatment for mUC.

**Table 4:** Previous reports concerning the association between proton pump inhibitors or potassium-competitive acid blocker use and immune checkpoint inhibitor response.

References	Generic name of PPI/P-CAB	Number of Pts, (Y/N)	Country or region	ICI	Cancer type	Endpoint (result of outcome)
Mukherjee et al.^9^	not mentioned	158 (73/85)	USA	Anti–PD-1 (pembrolizumab, REGN2810), anti–PD-L1 (nivolumab), others	All malignancies	PFS (NS)
Spakowicz et al.^10^	not mentioned	689 (255/434)	USA	Anti–PD-1 (pembrolizumab, durvalumab), anti–PD-L1 (atezolizumab, nivolumab), anti–CTLA-4 (ipilimumab, tremelimumab)	All malignancies	OS (NS)
Buti et al.^11^	not mentioned	217 (104/113)	USA	Anti–PD-1, anti–PD-L1, anti–CTLA-4	All malignancies	OS (worse) PFS (worse)
Chalabi et al.^12^	omeprazole, pantoprazole, lansoprazole, rabeprazole, esomeprazole, dexlansoprazole	757 (169/588)	Worldwide	Anti–PD-L1 (atezolizumab)	NSCLC	OS (worse) PFS (worse)
Hopkins et al.^13^	omeprazole, pantoprazole, lansoprazole, rabeprazole, esomeprazole, dexlansoprazole	1202 (441/761)	Worldwide	Anti–PD-L1 (atezolizumab)	NSCLC	OS (worse)
Hopkins et al.^14^	omeprazole, pantoprazole, lansoprazole, rabeprazole, esomeprazole, dexlansoprazole	895 (286/609)	Europe, North America and the Asia–Pacific region	Anti–PD-L1 (atezolizumab)	mUC	ORR (worse) OS (worse) PFS (worse)
Ruiz-Banobre et al.^15^	not mentioned	119 (54/65)	Spain	Anti–PD-1 (pembrolizumab, durvalumab), anti–PD-L1 (atezolizumab, nivolumab)	mUC	ORR (worse) OS (worse) PFS (worse) DCR (worse)

CTLA, cytotoxic T-lymphocyte–associated protein 4; DCR, disease control rate; ICI, immune checkpoint inhibitors; mUC, metastatic urothelial carcinoma; NS, not significant; NSCLC, non-small-cell lung cancer; ORR, objective response rate; OS, overall survival; PD-1, programmed cell death-1; PD-L1, programmed death-ligand 1; PFS, progression-free survival; PPI/P-CAB, proton pump inhibitor or potassium-competitive acid blocker; Pts, patients.

Several limitations were noted. First, this study was a retrospective multicenter analysis; therefore, it had shortcomings such as small sample sizes and a selection bias. Second, enrolled patients were almost all Japanese. In this regard, therefore, it is important to note that the distribution of the gut microbiome differs between ethnicities, regions, and diets^23^. Finally, we did not investigate a change in the gut microbiome of our patients even though dysbiosis generated by PPI has been shown in experimental animals and healthy humans. Any differences in the gut microbiome between patients with mUC and healthy humans, or dysbiosis induced by PPI/P-CAB in patients with mUC, remain to be elucidated.

In conclusion, of the gut microbiome–altering drugs examined, PPI/P-CAB had a negative influence on pembrolizumab used against mUC with respect to disease control, suggesting that the use of PPI/P-CAB should be carefully evaluated. Further investigations of the gut microbiome of patients with mUC treated with ICI and gut microbiome–altering drugs are needed.

### Supplementary Information

Supplementary Table 1.

Supplementary Table 2.

Supplementary Table 3.

Supplementary Table 4.

## Data Availability

The datasets analyzed and/or generated during the current study are not publicly available. However, they are available from the corresponding author after reasonable request.

## Abbreviations

AEs: Adverse events
CT: Computer tomography
CTCAE: Common Terminology Criteria for Adverse Events
ECOG: Eastern Cooperative Oncology Group
ICI: Immune checkpoint inhibitors
mAb: Monoclonal antibodies
mUC: Metastatic urothelial carcinoma
NLR: Neutrophil-to-lymphocyte ratio
NSAIDs: Non-steroidal anti-inflammatory drugs
OS: Overall survival
P-CAB: Potassium-competitive acid blockers
PFS: Progression-free survival
PPI: Proton pump inhibitors
PS: Performance status
RECIST: Response Evaluation Criteria in Solid Tumors
