# Supplementary material for: Proton pump inhibitors and potassium competitive acid blockers decrease pembrolizumab efficacy in patients with metastatic urothelial carcinoma
Source: Sci Rep. 2024 Jan 30;14:2520. doi: 10.1038/s41598-024-53158-1 (PMC10827730; doi:10.1038/s41598-024-53158-1)
Supplement: Supplementary file 1 — Supplementary Table 1. [file 41598_2024_53158_MOESM1_ESM.docx]

| **Characteristics** | | **non- PPI/P-CAB user group**  **(n=82)** | **PPI user group**  **(n=37)** | **P-CAB user group**  **(n=14)** | ***p* value** |
| --- | --- | --- | --- | --- | --- |
| **Median age, years (range)** | | 72  (39–85) | 73  (48–87) | 75  (62–85) | 0.577 |
| **Gender, n (%)** | **Male** | 69 (84) | 24 (65) | 10 (71) | 0.052 |
|  | **Female** | 13 (16) | 13 (35) | 4 (29) |  |
| **Primary site, n (%)** | **Bladder** | 40 (49) | 16 (43) | 6 (43) | 0.591 |
|  | **Upper urinary tract** | 32 (39) | 18 (49) | 8 (57) |  |
|  | **Both** | 10 (12) | 3 (8) | 0 (0) |  |
| **Treatment lines**  **of ICI, n (%)** | **2^nd^ line** | 61 (74) | 25 (68) | 8 (57) | 0.332 |
|  | **3^rd^ line later** | 21 (26) | 12 (32) | 6 (43) |  |
| **ECOG**–**PS, n (%)** | **0, 1** | 66 (80) | 31 (84) | 9 (64) | 0.285 |
|  | **≥ 2** | 16 (20) | 6 (16) | 5 (36) |  |
| **Metastatic site, n (%)** | **Lymph node only** | 24 (29) | 11 (30) | 1 (7) | 0.351 |
|  | **Existence of liver metastasis** | 16 (20) | 10 (27) | 4 (29) |  |
|  | **Other** | 42 (51) | 16 (43) | 9 (64) |  |
| **H2 blockers, n (%)** | | 6 (7) | 1 (3) | 0 (0) | 0.500 |
| **Antibiotics, n (%)** | | 19 (22) | 10 (27) | 6 (43) | 0.295 |
| **NSAIDs, n (%)** | | 14 (16) | 5 (14) | 1 (7) | 0.819 |
| **Metformin, n (%)** | | 4 (5) | 0 (0) | 0 (0) | 0.559 |
| **Antipsychotics, n (%)** | | 4 (5) | 4 (11) | 0 (0) | 0.381 |
| **Steroids, n (%)** | | 3 (3) | 5 (14) | 3 (21) | 0.030 |
| **Opioids, n (%)** | | 11 (13) | 15 (41) | 3 (21) | 0.004 |
| **Median NLR levels, (range)** | | 3.1 (0.8–26.8) | 3.4 (1.0–15.9) | 4.3 (0.7–27.6) | 0.100 |
| **Median albumin level, g/dL (range)** | | 3.7 (2.2–4.7) | 3.5 (2.1–4.4) | 3.6 (2.2–4.4) | 0.354 |
| **Median Hb levels, g/dL (range)** | | 10.8 (7.0–15.8) | 9.9 (7.7–15.9) | 10.2 (7.4–14.2) | 0.045 |

**Supplementary Table 1 The correlation between gut microbiome–altering drugs and several known prognostic factors in the treatment of metastatic urothelial carcinoma with pembrolizumab.** *P*-values were evaluated using Fisher's exact test or a Mann–Whitney *U* test. ECOG-PS, Eastern Cooperative Oncology Group Performance Status; Hb, hemoglobin; ICI, immune checkpoint inhibitors; NLR, neutrophil-to-lymphocyte ratio; NSAIDs, non-steroidal anti-inflammatory drugs; PPI/P-CAB, proton pump inhibitors/potassium-competitive acid blockers
