# Supplementary material for: Proton pump inhibitors and potassium competitive acid blockers decrease pembrolizumab efficacy in patients with metastatic urothelial carcinoma
Source: Sci Rep. 2024 Jan 30;14:2520. doi: 10.1038/s41598-024-53158-1 (PMC10827730; doi:10.1038/s41598-024-53158-1)
Supplement: Supplementary file 2 — Supplementary Table 2. [file 41598_2024_53158_MOESM2_ESM.docx]

**Supplementary Table 2**

**Uni- and multivariate analyses predicting progression-free survival in patients with metastatic urothelial carcinoma treated with pembrolizumab as second-line treatment. Three groups (no PPI/P-CAB [none], PPI, P-CAB) were analyzed.**

| **Parameters** | **Univariate** | | |  | **Multivariate** | | |
| --- | --- | --- | --- | --- | --- | --- | --- |
|  | **HR** | **95% CI** | ***p* value** |  | **HR** | **95% CI** | ***p* value** |
| **Age at initiation of treatment** | 1.00 | 0.98–1.02 | 0.916 |  | - | - | - |
| **Gender,**  **female vs. male** | 1.23 | 0.77–1.97 | 0.380 |  | - | - | - |
| **Primary site, UTUC vs. bladder** | 1.11 | 0.83–1.50 | 0.463 |  | - | - | - |
| **ECOG-PS, 2 vs. 0, 1** | 2.91 | 1.86–4.53 | < 0.001 |  | 2.32 | 1.38–3.94 | 0.002 |
| **Treatment lines of ICI, 3^rd^ line later vs. 2^nd^ line** | 1.16 | 0.76–1.77 | 0.488 |  | - | - | - |
| **Liver metastasis, yes vs. no** | 2.39 | 1.53–3.73 | < 0.001 |  | 1.92 | 1.16–3.19 | 0.011 |
| **PPI/P-CAB** | - | - | - |  | - | - | - |
| **None** | Ref. | - | - |  | Ref. | - | - |
| **PPI** | 1.97 | 1.28–3.03 | 0.002 |  | 1.74 | 1.10–2.75 | 0.018 |
| **P-CAB** | 2.36 | 1.27–4.42 | 0.007 |  | 1.64 | 0.85–3.16 | 0.137 |
| **H2 blockers** | 1.39 | 0.61–3.17 | 0.439 |  | - | - | - |
| **Antibiotics** | 1.35 | 0.88–2.10 | 0.173 |  | - | - | - |
| **NSAIDs** | 0.99 | 0.60–1.66 | 0.982 |  | - | - | - |
| **Metformin** | 1.66 | 0.52–5.26 | 0.389 |  | - | - | - |
| **Antipsychotics** | 1.42 | 0.62–3.26 | 0.403 |  | - | - | - |
| **Steroids** | 3.47 | 1.82–6.62 | <0.001 |  | 1.63 | 0.79–3.37 | 0.191 |
| **Opioids** | 2.39 | 1.54–3.72 | <0.001 |  | 1.29 | 0.80–2.08 | 0.300 |
| **NLR, ≥ 3.0 vs. < 3.0** | 1.97 | 1.31–2.96 | 0.001 |  | 1.45 | 0.92–2.27 | 0.110 |
| **Serum Alb levels** | 0.52 | 0.37–0.72 | <0.001 |  | 0.68 | 0.44–1.04 | 0.078 |
| **Hb levels** | 0.87 | 0.78–0.98 | 0.018 |  | 0.89 | 0.77–1.03 | 0.125 |

Alb, albumin; CI, confidence interval; ECOG-PS, Eastern Cooperative Oncology Group Performance Status; Hb, hemoglobin; HR, hazard ratio; ICI, immune checkpoint inhibitors; NLR, neutrophil-to-lymphocyte ratio; NSAIDs, non-steroidal anti-inflammatory drugs; PPI/P-CAB, proton pump inhibitors/potassium-competitive acid blockers; UTUC, upper urinary tract urothelial carcinoma
