# Supplementary material for: Proton pump inhibitors and potassium competitive acid blockers decrease pembrolizumab efficacy in patients with metastatic urothelial carcinoma
Source: Sci Rep. 2024 Jan 30;14:2520. doi: 10.1038/s41598-024-53158-1 (PMC10827730; doi:10.1038/s41598-024-53158-1)
Supplement: Supplementary file 3 — Supplementary Table 3. [file 41598_2024_53158_MOESM3_ESM.docx]

**Supplementary Table 3**

| **Parameters** | **Univariate** | | |  | **Multivariate** | | |
| --- | --- | --- | --- | --- | --- | --- | --- |
|  | **HR** | **95% CI** | ***p* value** |  | **HR** | **95% CI** | ***p* value** |
| **Age at initiation of treatment** | 1.01 | 0.99–1.03 | 0.433 |  | - | - | - |
| **Gender,**  **female vs. male** | 0.87 | 0.51–1.51 | 0.625 |  | - | - | - |
| **Primary site, UTUC vs. bladder** | 0.88 | 0.62–1.26 | 0.501 |  | - | - | - |
| **ECOG-PS, 2 vs. 0, 1** | 5.19 | 3.20–8.40 | < 0.001 |  | 3.83 | 2.15–6.81 | < 0.001 |
| **Treatment lines of ICI, 3^rd^ line later vs. 2^nd^ line** | 1.11 | 0.69–1.79 | 0.674 |  | - | - | - |
| **Liver metastasis, yes vs. no** | 3.34 | 2.05–5.43 | < 0.001 |  | 2.55 | 1.47–4.43 | < 0.001 |
| **PPI/P-CAB** | - | - | - |  | - | - | - |
| **None** | Ref. | - | - |  | Ref. | - | - |
| **PPI** | 1.47 | 0.89–2.45 | 0.134 |  | 1.36 | 0.79–2.35 | 0.272 |
| **P-CAB** | 2.23 | 1.11–4.47 | 0.024 |  | 1.46 | 0.68–3.13 | 0.327 |
| **H2 blockers** | 2.06 | 0.89–4.77 | 0.093 |  | - | - | - |
| **Antibiotics** | 1.43 | 0.86–2.36 | 0.168 |  | - | - | - |
| **NSAIDs** | 1.25 | 0.71–2.20 | 0.444 |  | - | - | - |
| **Metformin** | 1.24 | 0.39–3.98 | 0.715 |  | - | - | - |
| **Antipsychotics** | 1.16 | 0.36–3.70 | 0.805 |  | - | - | - |
| **Steroids** | 3.85 | 1.88–7.88 | <0.001 |  | 1.18 | 0.52–2.66 | 0.699 |
| **Opioids** | 3.79 | 2.32–6.19 | <0.001 |  | 1.97 | 1.12–3.49 | 0.019 |
| **NLR, ≥ 3.0 vs. < 3.0** | 2.10 | 1.31–3.37 | 0.002 |  | 1.15 | 0.68–1.97 | 0.602 |
| **Serum Alb levels** | 0.37 | 0.25–0.54 | < 0.001 |  | 0.40 | 0.24–0.67 | <0.001 |
| **Hb levels** | 0.85 | 0.75–0.97 | 0.019 |  | 0.91 | 0.76–1.09 | 0.308 |

**Uni- and multivariate analyses predicting overall survival for patients with metastatic urothelial carcinoma treated with pembrolizumab as second-line treatment. Three groups (no PPI/P-CAB [none], PPI, P-CAB) were analyzed.**

Alb, albumin; CI, confidence interval; ECOG-PS, Eastern Cooperative Oncology Group Performance Status; Hb, hemoglobin; HR, hazard ratio; ICI, immune checkpoint inhibitors; NLR, neutrophil-to-lymphocyte ratio; NSAIDs, non-steroidal anti-inflammatory drugs; PPI/P-CAB, proton pump inhibitors/potassium-competitive acid blockers; UTUC, upper urinary tract urothelial carcinoma
