# Supplementary material for: Proton pump inhibitors and potassium competitive acid blockers decrease pembrolizumab efficacy in patients with metastatic urothelial carcinoma
Source: Sci Rep. 2024 Jan 30;14:2520. doi: 10.1038/s41598-024-53158-1 (PMC10827730; doi:10.1038/s41598-024-53158-1)
Supplement: Supplementary file 4 — Supplementary Table 4. [file 41598_2024_53158_MOESM4_ESM.docx]

| **Parameters** | **Univariate** | | |  | **Multivariate** | | |
| --- | --- | --- | --- | --- | --- | --- | --- |
|  | **HR** | **95% CI** | ***p* value** |  | **HR** | **95% CI** | ***p* value** |
| **Age at initiation of treatment** | 1.00 | 0.98–1.02 | 0.916 |  | - | - | - |
| **Gender,**  **female vs. male** | 1.23 | 0.77–1.97 | 0.380 |  | - | - | - |
| **Primary site, UTUC vs. bladder** | 1.11 | 0.83–1.50 | 0.463 |  | - | - | - |
| **ECOG-PS, 2 vs. 0, 1** | 2.91 | 1.86–4.53 | < 0.001 |  | 2.17 | 1.30–3.63 | 0.003 |
| **Treatment lines of ICI, 3^rd^ line later vs. 2^nd^ line** | 1.16 | 0.76–1.77 | 0.488 |  | - | - | - |
| **Liver metastasis, yes vs. no** | 2.39 | 1.53–3.73 | < 0.001 |  | 2.05 | 1.23–3.41 | 0.006 |
| **PPI/P-CAB** | 2.06 | 1.39–3.06 | < 0.001 |  | 1.71 | 1.13–2.57 | 0.011 |
| **H2 blockers** | 1.39 | 0.61–3.17 | 0.439 |  | - | - | - |
| **Antibiotics** | 2.13 | 1.16–3.91 | 0.014 |  | 1.85 | 0.96–3.56 | 0.064 |
| **NSAIDs** | 0.99 | 0.60–1.66 | 0.982 |  | - | - | - |
| **Metformin** | 1.66 | 0.52–5.26 | 0.389 |  | - | - | - |
| **Antipsychotics** | 1.42 | 0.62–3.26 | 0.403 |  | - | - | - |
| **Steroids** | 3.47 | 1.82-6.62 | <0.001 |  | 1.41 | 0.67–2.99 | 0.367 |
| **Opioids** | 2.39 | 1.54-3.72 | <0.001 |  | 1.39 | 0.87–2.23 | 0.168 |
| **NLR, ≥ 3.0 vs. < 3.0** | 1.97 | 1.31–2.96 | 0.001 |  | 1.47 | 0.93–2.31 | 0.098 |
| **Serum Alb levels** | 0.52 | 0.37–0.72 | <0.001 |  | 0.68 | 0.44–1.04 | 0.074 |
| **Hb levels** | 0.87 | 0.78–0.98 | 0.018 |  | 0.90 | 0.78-1.04 | 0.153 |

**Supplementary Table 4**

**Sub-analysis of the effect of antibiotic use for a long period (more than 8 days) on predicting disease progression (A) and death (B) in patients with metastatic urothelial carcinoma and pembrolizumab as second-line treatment.**

Alb, albumin; CI, confidence interval; ECOG-PS, Eastern Cooperative Oncology Group Performance Status; Hb, hemoglobin; HR, hazard ratio; ICI, immune checkpoint inhibitors; NLR, neutrophil-to-lymphocyte ratio; NSAIDs, non-steroidal anti-inflammatory drugs; PPI/P-CAB, proton pump inhibitor/potassium-competitive acid blocker; UTUC, upper urinary tract urothelial carcinoma

**(A)**

| **Parameters** | **Univariate** | | |  | **Multivariate** | | |
| --- | --- | --- | --- | --- | --- | --- | --- |
|  | **HR** | **95% CI** | ***p* value** |  | **HR** | **95% CI** | ***p* value** |
| **Age at initiation of treatment** | 1.01 | 0.99–1.03 | 0.433 |  | - | - | - |
| **Gender,**  **female vs. male** | 0.87 | 0.51–1.51 | 0.625 |  | - | - | - |
| **Primary site, UTUC vs. bladder** | 0.88 | 0.62–1.26 | 0.501 |  | - | - | - |
| **ECOG-PS, 2 vs. 0, 1** | 5.19 | 3.20–8.40 | < 0.001 |  | 3.66 | 2.07–6.47 | < 0.001 |
| **Treatment lines of ICI, 3^rd^ line later vs. 2^nd^ line** | 1.11 | 0.69–1.79 | 0.674 |  | - | - | - |
| **Liver metastasis, yes vs. no** | 3.34 | 2.05–5.43 | < 0.001 |  | 2.67 | 1.53–4.66 | < 0.001 |
| **PPI/P-CAB** | 1.64 | 1.04–2.59 | 0.033 |  | 1.37 | 0.84–2.22 | 0.205 |
| **H2 blockers** | 2.06 | 0.89–4.77 | 0.093 |  | - | - | - |
| **Antibiotics** | 2.04 | 1.01–4.10 | 0.047 |  | 1.96 | 0.90–4.27 | 0.089 |
| **NSAIDs** | 1.25 | 0.71–2.20 | 0.444 |  | - | - | - |
| **Metformin** | 1.24 | 0.39–3.98 | 0.715 |  | - | - | - |
| **Antipsychotics** | 1.16 | 0.36–3.70 | 0.805 |  | - | - | - |
| **Steroids** | 3.85 | 1.88-7.88 | <0.001 |  | 1.01 | 0.43–2.34 | 0.986 |
| **Opioids** | 3.79 | 2.32-6.19 | <0.001 |  | 2.18 | 1.26–3.76 | 0.005 |
| **NLR, ≥ 3.0 vs. < 3.0** | 2.10 | 1.31–3.37 | 0.002 |  | 1.15 | 0.67–1.97 | 0.607 |
| **Serum Alb levels** | 0.37 | 0.25–0.54 | < 0.001 |  | 0.38 | 0.22–0.64 | <0.001 |
| **Hb levels** | 0.85 | 0.75–0.97 | 0.019 |  | 0.93 | 0.77-1.12 | 0.436 |

**(B)**
